# Lipid Interactions Between Flaviviruses and Mosquito Vectors

**DOI:** 10.3389/fphys.2021.763195

**Published:** 2021-11-05

**Authors:** Thomas Vial, Guillaume Marti, Dorothée Missé, Julien Pompon

**Affiliations:** ^1^Programme in Emerging Infectious Diseases, Duke-NUS Medical School, Singapore, Singapore; ^2^UMR 152 PHARMADEV-IRD, Université Paul Sabatier, Toulouse, France; ^3^LRSV (UMR 5546), CNRS, Université de Toulouse, Toulouse, France; ^4^MetaboHUB, National Infrastructure of Metabolomics and Fluxomics, Toulouse, France; ^5^MIVEGEC, Université Montpellier, IRD, CNRS, Montpellier, France

**Keywords:** mosquito, flavivirus, phospholipids, transmission, metabolomics

## Abstract

Mosquito-borne flaviviruses, such as dengue (DENV), Zika (ZIKV), yellow fever (YFV), West Nile (WNV), and Japanese encephalitis (JEV) viruses, threaten a large part of the human populations. In absence of therapeutics and effective vaccines against each flaviviruses, targeting viral metabolic requirements in mosquitoes may hold the key to new intervention strategies. Development of metabolomics in the last decade opened a new field of research: mosquito metabolomics. It is now clear that flaviviruses rely on mosquito lipids, especially phospholipids, for their cellular cycle and propagation. Here, we review the biosyntheses of, biochemical properties of and flaviviral interactions with mosquito phospholipids. Phospholipids are structural lipids with a polar headgroup and apolar acyl chains, enabling the formation of lipid bilayer that form plasma- and endomembranes. Phospholipids are mostly synthesized through the *de novo* pathway and remodeling cycle. Variations in headgroup and acyl chains influence phospholipid physicochemical properties and consequently the membrane behavior. Flaviviruses interact with cellular membranes at every step of their cellular cycle. Recent evidence demonstrates that flaviviruses reconfigure the phospholipidome in mosquitoes by regulating phospholipid syntheses to increase virus multiplication. Identifying the phospholipids involved and understanding how flaviviruses regulate these in mosquitoes is required to design new interventions.

## Mosquito-Transmitted Flaviviruses

### Global Pathogens

Flaviviruses like dengue (DENV), Zika (ZIKV), yellow fever (YFV), West Nile (WNV), and Japanese encephalitis (JEV) viruses threaten almost the whole human population (Pierson and Diamond, 2020). They cause half a billion infections per year that result in about 250,000 deaths and an economic loss of $8.9 billion. All these viruses are transmitted through the bite of mosquito vectors, which geographic distributions are steadily increasing because of global changes. As a result, these diseases that were once restricted to the tropics have now encroached on temperate regions, encompassing new immunologically naïve populations. More alarmingly, the emergence of ZIKV and WNV in the last decades suggests that one should expect other flaviviruses to emerge as epidemic in the near future (Pierson and Diamond, 2020).

There is no effective means to control all flaviviruses (Barrows et al., 2018). Rare examples of sustained vector control showed its ineffectiveness to prevent epidemics, even in small isolated urbanized areas (Ooi et al., 2006). The design of safe vaccines is challenging. Indeed, some antibodies generated during a primary flavivirus infection cross-react with a secondary heterologous flavivirus infection but fail to neutralize the second infection. These cross-reacting non-neutralizing antibodies then facilitate attachment and cell entry of the second virus, increasing infection and aggravating symptoms (Halstead, 2007) (a phenomenon called Antibody-Dependent Enhancement, ADE). The only licensed vaccine against DENV (i.e., DENGVAXIA) suffers from this limitation as it can increase dengue symptom severity in dengue-naïve patients (Halstead, 2017; Shukla et al., 2020; Huang et al., 2021). Moreover, there is no therapeutics and treatments are limited to supportive care.

Recent technical developments enabled the study of metabolic interactions in flavivirus infection [methodology of metabolomics was extensively reviewed in Nunes and Canuto (2020)]. Multiple evidence demonstrates the roles of lipids in infections in both vertebrate hosts and vectors (Leier et al., 2018; Byers et al., 2019; O’Neal et al., 2020), although the molecular mechanisms that orchestrate the lipidome modulations remain mostly elusive. Characterized lipid factors are potential targets for new mosquito-targeted interventions (Lu and Chen, 2017; Manchester and Anand, 2017). In this review, we focus on the biochemistry and functions of lipid metabolism in flavivirus infection in mosquito vectors.

### Flaviviruses

**FIGURE 1:**
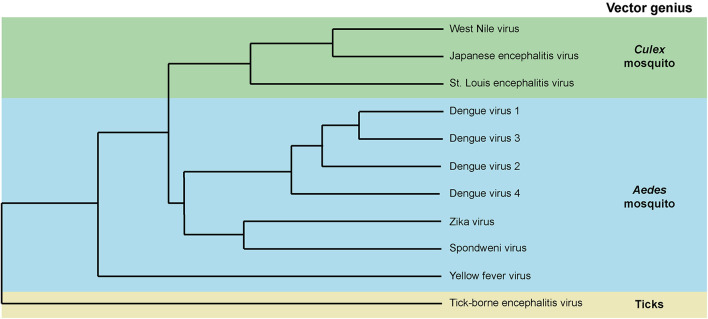
Phylogenetic distribution of mosquito-borne flaviviruses and their associated vectors. Dendrogram based on amino acid sequences.

Mosquito-transmitted flaviviruses such as the four DENV serotypes (DENV 1–4), ZIKV, YFV, JEV, and WNV are genetically related and primarily transmitted by either *Aedes* or *Culex* mosquitoes (Alkan et al., 2015; Lazear and Diamond, 2016; Figure 1). Flaviviruses are enveloped viruses with a spherical shape of about 50 nm diameter. The envelope is composed of two proteins, the Envelope and Membrane, which are anchored in a lipid bilayer. Within the envelope structure, a single-stranded positive-sense RNA genome of about 11 kb is packaged in association with the capsid protein. The genome codes for three structural proteins – Envelope, pr-Membrane, and Capsid – and seven non-structural proteins – NS1, NS2A, NS2B, NS3, NS4A, NS4B, and NS5. In the virion, the lipid bilayer is the only component that is not encoded in the viral genome and derives from the vector.

### Mosquito Transmission

Viruses collected during a blood meal on an infected host first infect the mosquito midgut epithelium (Salazar et al., 2007). While blood meal is digested in 48 h, viral replication in the midgut continues and reaches a peak at 7 days after blood ingestion. From the midgut, viruses disseminate in the whole mosquito body, including salivary glands which are fully infected at 10–14 days post infectious blood meal. Mosquitoes can then transmit the viruses via expectorated saliva during subsequent bites.

Throughout infection in mosquito, flaviviruses are confronted to different barriers (Franz et al., 2015). After ingestion with blood, flaviviruses need to overpass an extracellular matrix to infect the midgut and then escape from the midgut to infect secondary tissues, such as hemocytes, fat body, and nerve tissues (Parikh et al., 2009). Viruses eventually infect lateral and median lobes of salivary glands (Salazar et al., 2007), which produce viral particles released in the excretory canal during biting. The ability of a mosquito to acquire and propagate viruses through all these steps is defined as vector competence. Vector competence is related to physical tissue barriers and genetic factors which, among others, determine immune response (Hardy et al., 1983). Because of the virus requirements for lipids, cellular lipid composition is another important factor that determines vector competence.

## Phospholipid: A Structural Lipid on the Frontline

### Phospholipid Definition

**FIGURE 2:**
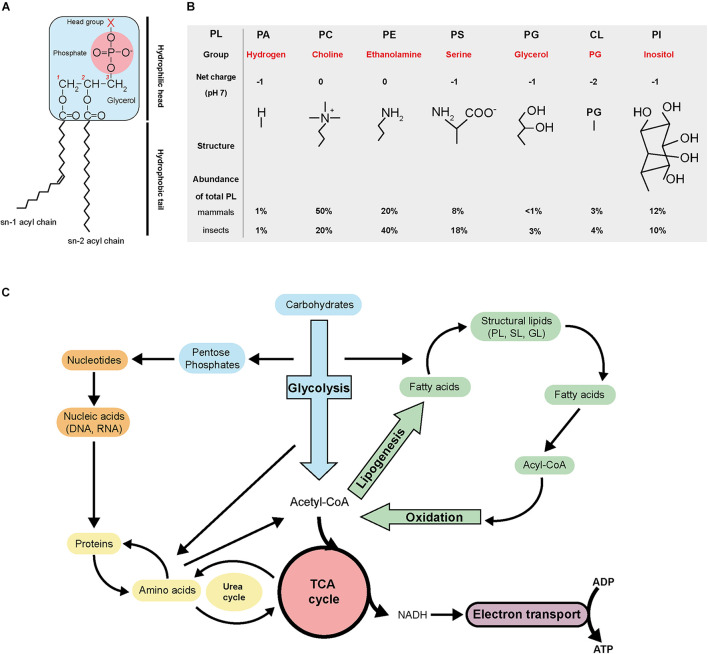
Phospholipid structure and general metabolism. **(A)** Schematic of phospholipid structure. **(B)** Basic characteristics of the different phospholipid categories. **(C)** Overview of the general metabolism. PL, phospholipid; PA, phosphatidic acid; PC, phosphatidylcholine; PE, phosphatidylethanolamine; PS, phosphatidylserine; PG, phosphatidylglycerol; CL, cardiolipin; PI, phosphatidylinositol; TCA, tricarboxylic cycle; SL, sphingolipid; GL, glycerolipid; NADH, nicotinamide adenine dinucleotide; ADP, adenosine diphosphate; ATP, adenosine triphosphate.

Phospholipids (PL) are composed of one hydrophilic head group, a glycerol backbone and two hydrophobic fatty acyl chains (Figure 2A). Depending on their head group, they are categorized as phosphatidic acid (PA), phosphatidylcholine (PC), phosphatidylethanolamine (PE), phosphatidylserine (PS), phosphatidylglycerol (PG), cardiolipin (CL), or phosphatidylinositol (PI; van Meer et al., 2008; Figure 2B). The amphiphilic (i.e., having both hydrophilic and hydrophobic ends) nature of PL enables the formation of bilayers with the hydrophobic acyl chain turned inward. PLs are the major structural lipids and generate physical barriers circumventing cellular contents and segregating cytosolic compartments, enabling intracellular organelle formation and compartmentalization of different cellular activities (Vance, 2015).

Phospholipid biosynthesis is intimately connected with the tricarboxylic (TCA) cycle and glycolysis (Figure 2C). Carbohydrate catabolism during glycolysis, fatty acids oxidation and amino acid recycling produce acetyl-CoA. Acetyl-CoA then serves as a precursor for biosynthesis of fatty acids via lipogenesis. Acetyl-CoA also feeds in the TCA cycle to produce precursors of amino acids and the reducing agent NADH, which goes into the electron transport chain to produce chemical energy in the form of ATP. The pentose phosphate pathway derived from glycolysis generates nucleotides and nucleic acids. All the general metabolic pathways are interconnected.

### Fatty Acid Biosynthesis

**FIGURE 3:**
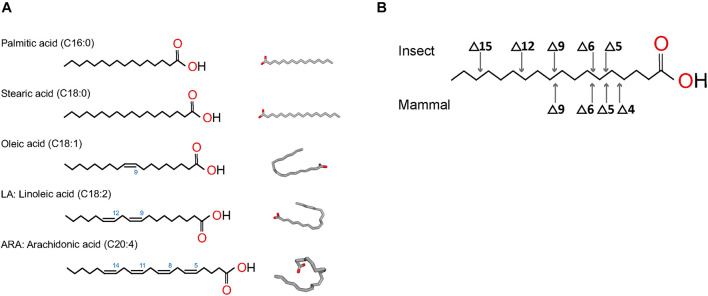
Structures of most common fatty acids found in phospholipids. **(A)** Fatty acids saturated and unsaturated are represented in linear form and 2D conformation from https://pubchem.ncbi.nlm.nih.gov. **(B)** Desaturation positions in fatty acyls in insects and mammals.

The fatty acyl chains of PL can have different carbon number and degree of saturation, both of which are generated during fatty acid biosynthesis. Fatty acid synthesis starts with the formation of malonyl-CoA from acetyl-CoA, via acetyl-CoA carboxylase (Sul and Smith, 2008). In the cytoplasm, fatty acid synthase then catalyzes repeated additions of acetyl-CoA to mainly produce palmitic acid (palmitate), a 16-carbon saturated fatty acid (C16:0), and in minor amount, a 18-carbon stearic acid (C18:0) (Figure 3A). Palmitic or stearic acids undergo elongation or unsaturation to generate other types of fatty acids, such as oleic acid, linoleic acid, or arachidonic acid (Figure 3A; Cook and McMaster, 2002). Chain elongation is happening mainly in the endoplasmic reticulum (ER) and produces acyl chains greater than 16 carbons, by successive 2-carbon condensation from malonyl-CoA. Mitochondrial elongation is less important but is required for mitochondrial membrane biogenesis. Fatty acids are used as components of membrane lipids or can be esterified in triacylglycerol for energy storage.

Mono-unsaturated fatty acids are produced by oxidative desaturation by desaturases to form a double bond. The first double bond introduced is generally in carbon at position 9 (Δ9) (Figure 3B). In most mammals, poly-unsaturated fatty acids result from the insertion of other double bonds between the existing bond and the carboxyl end of the chain, but not on the methyl end (Figure 3B; Rivers et al., 1975). In humans, poly-unsaturated fatty acids, such as arachidonic acid, are synthetized through modification of linoleic acid via a Δ6/Δ5 desaturase, followed by elongation steps (Bond et al., 2016). Insects, however, can desaturate on either side of the existing bond in Δ9 thanks to specific desaturases (Cook and McMaster, 2002). As a result, insect poly-unsaturated fatty acids contain double-bonds in positions Δ5 to Δ15, whereas mammal fatty acids can only produce direct double-bonds in positions Δ4 to Δ9 (Figure 3B).

### *De novo* Phospholipid Biogenesis

**FIGURE 4:**
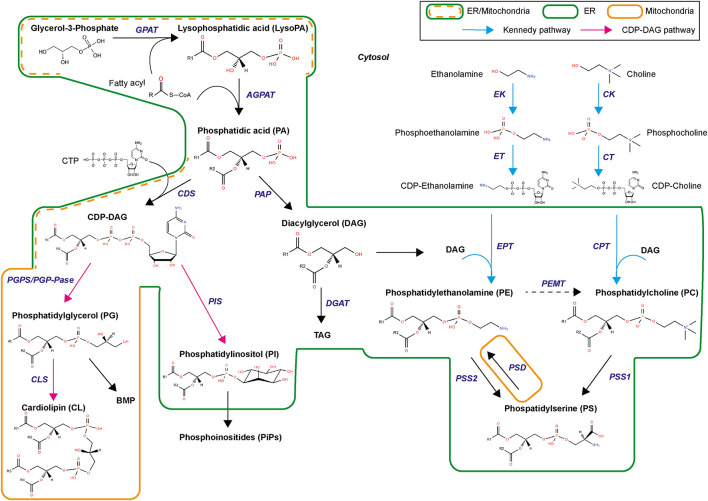
Biosynthetic pathways for phospholipids. GPAT, glycerol-3-phosphate acyltransferase; AGPAT, 1-acyl-sn-glycerol-3-phosphate O-acyltransferases; CDS, CDP-diacylglycerol synthase; PGP, PG phosphate synthase, CLS, cardiolipin synthase, PIS, PI synthase; PAP, PA phosphatase; DGAT, Diglyceride acyltransferase; EK, ethanolamine kinase; CK, choline kinase; ET, triphosphate:phosphoethanolamine cytidylyltransferase; CT, triphosphate:phosphocholine cytidylyltransferase; EPT, DAG:CDP-ethanolamine ethanolaminephosphotransferase; CPT, DAG:CDP-choline cholinephosphotransferase; PEMT, PE methyltransferase; PSD, PS decarboxylase; PSS1, PS synthase 1; PSS2, PS synthase 2.

Phospholipid biogenesis is highly conserved throughout the animal kingdom. It involves multiple enzymes in different organelles and results in the production of hundreds of different PL species (Figure 4). PL *de novo* biogenesis is initiated by two types of acyl-transferases that sequentially add two acyls to one glycerol-3-phosphate (Fagone and Jackowski, 2009; Takeuchi and Reue, 2009; Vance, 2015). The first addition is realized in the ER or mitochondria-associated membranes by glycerol-3-phosphate acyltransferase (GPAT) to produce lysoPA. LysoPAs produced in mitochondria are then transferred to the ER prior to the second acylation. The second addition is catalyzed by 1-acyl-sn-glycerol-3-phosphate O-acyltransferases (AGPAT) that transform lysoPA in PA in the ER principally (Yamashita et al., 2014). The new PA is composed of fatty acids linked to the glycerol molecule at the first and second carbons, called sn1 and sn2 positions, respectively. Fatty acids in sn1 are generally saturated or monounsaturated, while fatty acids in sn2 are polyunsaturated with longer chains (Beermann et al., 2005). The most abundant fatty acids in sn1 are palmitic acid (16:0), stearic acid (18:0), and oleic acid (18:1); and in sn2 are linoleic acid (18:2), arachidonic acid (20:4), eicosapentaenoic acid (20:5), or docosahexaenoic acid (22:6) (Figure 3A). The diversity of fatty acids results in a multiplicity of PA species, which are then used to produce all PLs. Due to their implication in generating PA, GPATs are rate-limiting enzymes in PL biogenesis (Yamashita et al., 2014).

*De novo* PL biogenesis then separates in two branches, producing either diacylglycerol (DAG) or cytidine diphosphate (CDP)-DAG (Figure 4). Synthesis of CDP-DAG involves condensation of cytidine triphosphate via CDP-DAG synthase. This reaction occurs in ER and mitochondria-associated membranes. CDP-DAG is used to produce PI and PG. PGs are synthesized in both ER and mitochondria by phosphatidylglycerol phosphate synthase and phosphatase. PG can produce CL by combination with CDP-DAG via cardiolipin synthase or bis(monoacylglycerol)phosphate via a complex biosynthetic pathway of acylations. PI is synthesized by condensation of CDP-DAG with inositol via PI synthase. Inositol originates from diet, recycling, or biosynthesis from glucose. PI is the precursor of several phosphorylated derivatives, also called phosphoinositides, which are involved in cell signaling.

Phosphatidic acid is also transformed in DAG by PA phosphatase (Figure 4). DAG produces triacylglycerol and feeds into the biosynthesis of aminophospholipids (aminoPL), namely PC, PE, and PS (Vance, 2015). *De novo* PC and PE synthesis is conducted through the Kennedy pathway (Gibellini and Smith, 2010) within the ER, using CDP-choline and CDP-ethanolamine intermediates. Choline and ethanolamine are first phosphorylated by choline/ethanolamine kinases (CK/EK). Phosphocholine and phosphoethanolamine then form CDP-choline and CDP-ethanolamine by combining with cytidine triphosphate through the rate-limiting enzyme cytidine triphosphate:phosphocholine/ethanolamine cytidylyltransferase (CT/ET). DAG incorporates the phosphocholine/phosphoethanolamine group from CDP-choline or CDP-ethanolamine with DAG:CDP-choline cholinephosphotransferase (CPT) or DAG:CDP-ethanolamine ethanolaminephosphotransferase (EPT) to produce PC and PE, respectively. Alternative pathways exist for PC and PE syntheses. PE biosynthesis through the PS decarboxylase (PSD) pathway in the mitochondria inner membrane involves PS decarboxylation into PE by a PS decarboxylase (Horvath et al., 2012; Tamura et al., 2012). PC can also be synthetized in a minor pathway via ethanolamine methylation of PE by PE methyltransferase, principally in hepatocytes. PC and PE are used to produce PS by head exchange reaction catalyzed by PS synthases (PSS1 or PSS2). Direct PS biosynthesis via CDP-DAG pathway was found in plants and yeast (Yamashita and Nikawa, 1997). PCs are also used in sphingomyelin synthesis by sphingomyelin synthase (SMS).

Given their roles in aminoPL biosynthesis, choline and ethanolamine are essential nutrients that need to be imported from the diet (Gottlieb et al., 1999). Alternatively, minor mechanisms of recycling exist (Vance et al., 2007; Vance and Vance, 2008; Vance, 2013). Methylation of PE into PC followed by hydrolysis can recycle choline, while small amount of ethanolamine can be produced by PE degradation. Intracellularly, although choline and ethanolamine are rapidly phosphorylated, incorporation into CDP is a limiting reaction (Wortmann and Mayr, 2019). Consequently, phosphocholine and phosphoethanolamine are in higher quantities than the product from the following step, CDP-choline and CDP-ethanolamine. There exist feed-back and feed-forward mechanisms to adjust aminoPL production based on PL requirements (Patton-Vogt and de Kroon, 2020).

### Phospholipid Remodeling

**FIGURE 5:**
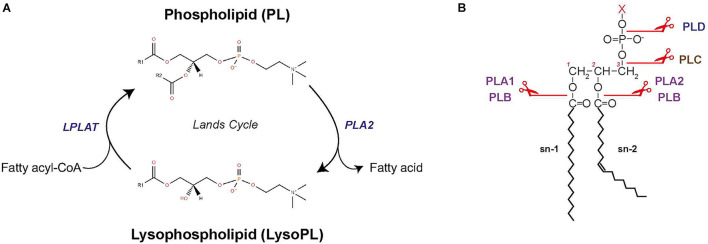
Phospholipid remodeling. **(A)** Schematic of PL remodeling through the Lands cycle. **(B)** Sites of cleavage by the different phospholipases. LPLAT, lysophospholipid acyltransferase; PLA1, phospholipase A1; PLA2, phospholipase A2; PLB, phospholipase B; PLC, phospholipase C; PLD, phospholipase D.

Phospholipid remodeling occurs through the Lands cycle and reconfigures fatty acyls in PLs, thereby increasing diversity of fatty acyls in *de novo* PLs (Wang and Tontonoz, 2019; Figure 5A). PLs are first hydrolyzed at the sn2 position by phospholipase A2 (PLA2) to produce 1-acyl lysoPL (Figure 5B). The lysoPL is then reacylated by lysophospholipid acyltransferase (LPLAT) via incorporation of another fatty acid in sn2 position, thereby forming a new PL species (Wang and Tontonoz, 2019). Remodeling enables modulation of PL membrane composition and cell signaling. For the latter, lysoPL and fatty acids released by PLA2 activity can serve as intermediates for synthesis of signaling lipids (O’Donnell et al., 2018).

Phospholipase A2 enzymes have multiple isoforms with different mechanisms of action (Burke and Dennis, 2009). Four main categories of PLA2 exist (Nor Aliza and Stanley, 1998; Abdul Rahim et al., 2018): (i) secreted PLA2, which is most studied in bees and snake venoms and found in pancreatic juices from mammals; (ii) cytosolic PLA2, which is recruited to the membrane by Ca^2+^-dependent translocation; (iii) calcium-independent PLA2; and (iv) platelet activating factor lipoprotein-associated PLA2. There exist other types of phospholipases that can hydrolyze PL (Figure 5B; Aloulou et al., 2018). PLA1 hydrolyzes the sn1 position to produce a fatty acid and a lysoPL. PLA1 function is largely unknown but likely related to production of lysoPS, lysoPI, and lysoPA. Phospholipase B (PLB) hydrolyzes both the sn1 and sn2 fatty acids of PC, PE, and PI (Morgan et al., 2004). Phospholipase C (PLC) and phospholipase D (PLD) are phosphodiesterase. PLC cleaves the glycerophosphate bond of PC and PI, producing DAG and a phosphorylated headgroup. PLD removes the headgroup to generate PA, but can also catalyze exchanges of headgroups by transphosphatidylation to produce new PL types. In mammals, PLD is involved in PA remodeling and can release choline when PCs are the substrate (Onono and Morris, 2020).

Among LPLAT (Figure 5A), lysophosphatidylcholine acyltransferases (LPCAT) were first discovered for catalyzing reacylation of lysoPC (Wang and Tontonoz, 2019). LPCATs actually have acyltransferase activity for lysoPE, lysoPS, and lysoPG. Four LPCATs were identified (LPCAT1–4) and each has specific substrate preference, enzymatic activity and tissue localization in mammals. LPCAT1 and LPCAT2 are members of the AGPAT family and are found in the ER membrane and lipid droplets. PL remodeling by LPCAT1–2 regulates the size and surface organization of lipid droplets (Moessinger et al., 2014). LPCAT3 and LPCAT4 are part of the membrane-bound O-acyltransferase family and are present in the ER membrane. LPCAT3 is the most expressed LPCAT in several cell types and is responsible for the bulk of lysoPC acyltransferase activity.

### Phospholipid Cellular Distribution

Endoplasmic reticulum is where the bulk of structural lipids (i.e., PL, ceramides, and cholesterols) are produced (Bell et al., 1981). As the first production organelle, the ER contains all intermediates and end products of complex lipid pathways, except for sterol and sphingolipids which are rapidly transported into other membranes. Mitochondria is also a major site of lipid biosynthesis, especially for lysoPA, PA, and PG used for CL synthesis, a product that is unique to this organelle. Mitochondria can produce PE by PS decarboxylation. The inner membrane of the mitochondria is composed of a high PG and CL content and a high PE/PC ratio (Horvath and Daum, 2013). A sub-fraction of the ER attached to the mitochondria, the mitochondria-associated membranes, contains specific enzymes for lipid biosynthesis (Rusiñol et al., 1994; Raturi and Simmen, 2013; Mayr, 2015). The Golgi is more specialized in sphingolipid production and the final steps of PC synthesis (Henneberry et al., 2002). Plasma membranes and early endosome contain more sterols and sphingolipids than PLs, due to the required property of resistance to mechanical stress. Plasma membrane is not a major place for structural lipid synthesis, even if lipid regulation occurs by sphingolipid turnover and lipid degradation (Di Paolo and De Camilli, 2006). Late endosome contains low concentrations of PS and sterol but a high concentration of bis(monoacylglycerol)phosphate, a lipid associated with fusion and sphingolipid degradation (Kobayashi et al., 2002; Kolter and Sandhoff, 2005).

### Phospholipid Biochemical Properties

#### Curvature of Membranes

**FIGURE 6:**
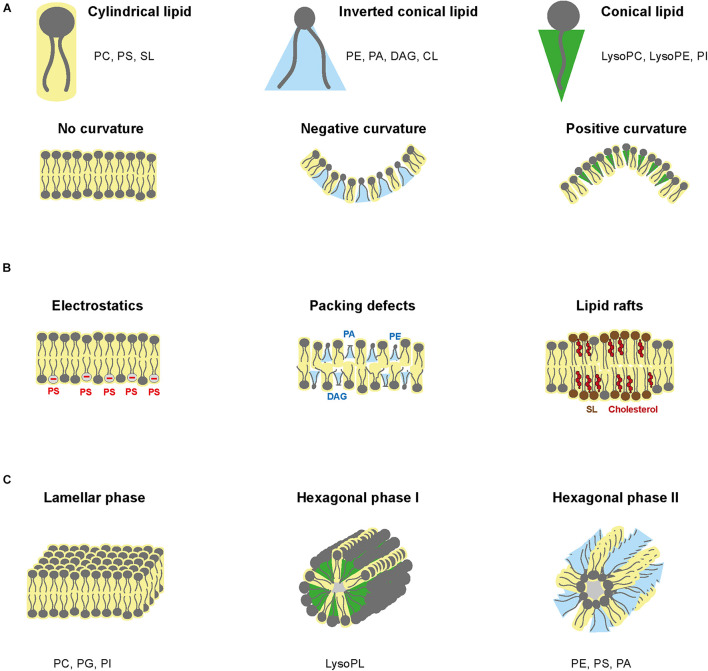
Phospholipid structure and biochemical properties. **(A)** Spatial hindrance of PLs and the implications for membrane shape. **(B)** PL influence on electrostatics, packing defects, and lipid rafts. **(C)** Schematic of lamellar phase, hexagonal phase type I and type II.

As steric hindrance of PL varies with the headgroup size and the acyl chains, PL composition influences membrane shape (Marsh, 2007; Marquardt et al., 2015; McMahon and Boucrot, 2015). PC and PS have a relatively large polar headgroup and parallel fatty acyl chains that confer a cylindrical geometry and enable linear bilayer formation (Figure 6A). Similarly, sphingolipids have a smaller headgroup and only one acyl chain, resulting in a cylindrical geometry. However, when inserted in bilayers, sphingolipids produce a tighter membrane because of their smaller steric hindrance. PE, PA, DAG, and CL have a small headgroup and two fatty acyls that form an inverted conical geometry (Figure 6A). When inserted in the inner layer of a lipid bilayer, these PL types impose a negative curvature (Marsh, 2007). Conversely, lysoPC, lysoPE, and PI have a thinner acyl chain hindrance, which confers positive curvature in lipid bilayer (Figure 6A).

#### Membrane Asymmetry

Membrane asymmetry refers to an asymmetric distribution of lipid species in lipid bilayers. Asymmetry induces biophysical properties that promote certain cellular functions (Fadeel and Xue, 2009; Marquardt et al., 2015). A well-known example is the organization of negatively charged PS in plasma membrane. In normal cells, PS are found on the cytoplasmic side where they associate with numerous enzymes such as kinases. During specific events, PS move to the outer leaflet, exposing their negative polar head to the extracellular side and inducing phagocytosis. In plasma membrane, PC and sphingolipid are generally found in the outer leaflet, while PE, PS, and PI are found in the inner leaflet (van Meer et al., 2008). Lipids in the ER are mostly symmetrically distributed between the two leaflets, while Golgi and endosome membranes have asymmetric distributions.

Phospholipid asymmetry is maintained by lipid transporters (Leventis and Grinstein, 2010; Kobayashi and Menon, 2018). Flippases are ATP-dependent aminoPL translocases that transport lipids inward. Floppases are ATP-binding cassette transporters that transport lipids outward. PL scramblases induce lipid asymmetry by exchanging lipids between the two membrane layers. For instance, PL scramblases induce PS externalization in apoptotic cells (Fadeel and Xue, 2009; Williamson, 2016).

#### Electrostatics

Electrostatic charges of membranes depend on negatively charged lipids, i.e., PS and phosphoinositides (PIPs) (Figure 6B). PS and PIPs are highly present in plasma membrane, especially on the cytosolic side and are in low abundance in ER membrane (Holthuis and Levine, 2005; Leventis and Grinstein, 2010). Furthermore, the charge of other PLs is pH-dependent. PC and PE are zwitterionic, whereas PS, PA, PG, CL, and PI are anionic at pH 7 (Li et al., 2015). Consequently, pH gradient modifies electric charges (Hope and Cullis, 1987). Electric charges are critical to orientate transmembrane proteins. Positively charged peptides will interact with negatively charged lipids in the inner leaflet to integrate into the lipid bilayer, while the protein position within the membrane is determined by charges from both lipid layers (Marquardt et al., 2015). Eventually, distribution of the membrane electric charges is influenced by lipid asymmetry.

#### Packing Defects

Lipid packing defects refer to heterogeneous lipid arrangements, which loosen lipid bilayer and increase fluidity, facilitating protein insertion (Janmey and Kinnunen, 2006). Ratio between small and large headgroups and ratio between saturated and unsaturated acyl chains influence lipid packing. Low packing in the ER is induced by high concentration of unsaturated PL and lack of cholesterol (Radhakrishnan et al., 2008), while plasma membrane has high packing due to saturated structural lipids and sterols (Figure 6B).

#### Lipid Phases

Lipid phase relates to the fluidity of lipid membranes and how lipids can move within each layer. The liquid phase is characterized by lateral mobility of lipids within their layer, whereas in the solid phase, also called gel phase, lipids lose lateral mobility. Lipid phase depends on the lipid composition and extrinsic factors such as temperature, pressure, and composition of the aqueous phase (van Meer et al., 2008). Most biomembranes are organized in lamellar (i.e., bilayer structure) with lipids in liquid phase and contain floating “rafts” of gel-phase lipids (Figure 6B). Lamellar structures are formed by non-curvature lipids such as PC, PG, and PI, while long and saturated chains, high amount of cholesterol and low temperature promote gel phase formation. The gel-phase rafts are enriched in saturated lipids but also sphingolipids and cholesterol, while the liquid-phase contains unsaturated PLs, which form kinks that loosen interactions with other lipids.

Membrane lipids can also form non-lamellar transitory structures, known as hexagonal phases (Jouhet, 2013; Figure 6C). Hexagonal phase can be of two types. Type I hexagonal phase has the polar head outside of the micelles, whereas type II consists of inverted micelles with fatty acyl chains directed outward. Combination of type II and lamellar phases can establish aqueous channels within the lipid bilayer. Non-lamellar phases exist temporarily during fusion, fission and pore formation and influences specific biochemical reactions (Jouhet, 2013). Negative curvature lipids, such as PE, PS, and PA, form hexagonal type II micelle phase, while positive curvature lipids such as lysoPL form hexagonal type I phase.

#### Protein Insertion

Proteins inserted in membranes drastically modify the behavior of lipid membranes. Insertion of proteins is influenced by the physicochemical parameters of the membrane, such as curvature, electrostatics, and lipid packing (Bigay and Antonny, 2012). Once inserted, the proteins perturb the hydrophobicity by provoking a mismatch between protein and lipid that affects the thickness and membrane organization (Janmey and Kinnunen, 2006). Inversely, protein function is also tightly regulated by lipid interactions, which can influence its interaction with other molecules (Lundbæk, 2006; Martens et al., 2018).

### Lipid Droplets: A Lipid Storage in Interaction With Phospholipids

Lipid droplets (LD) are essential for cell storage of carbons in the form of neutral lipids, triacylglycerol and sterol esters (Jackson, 2019). These storage bodies are constrained by a monolayer of PL (mainly PC and PE) and proteins (Bartz et al., 2007). LD are generated by neutral lipid accumulation (TAG and sterol esters) between the two ER phospholipid bilayers. Growing of this lipid lens produces a vesicle surrounded by a monolayer originating from the ER that is eventually released into the cytosol (Thiam and Forêt, 2016). LD biogenesis is tightly regulated by PL metabolism through GPAT, DGAT, and CPT enzymes, respectively, involved in LysoPA, TAG, and PC biosynthesis (Krahmer et al., 2011; Henne and Goodman, 2019). Other proteins not directly associated with lipid syntheses are involved in LD production and include seipin, perilipins and fat storage-inducing transmembrane (FIT) proteins. Nonetheless, seipin can regulate PL and neutral lipid synthesis, as well as PL transfer between the ER and LD (Wang et al., 2016; Yan et al., 2018).

Lipids in LD are mobilized by lipases to provide substrates for PL synthesis, fatty acid pathway, and signaling lipid production (Ducharme and Bickel, 2008). LD also contain enzymes of the PC biosynthesis pathway, allowing the production of CDP-choline (Penno et al., 2013). However, LD-produced CDP-choline is then transferred to the ER to complete PC production. Presence of PLA2 and LPCAT in LD can also influence PL remodeling and the PL monolayer surrounding LD. While this review focuses on the role of LD in PL metabolism, it should be noted that LD have other functions in vitamin storage, vitamin signaling, regulation of cellular stress, protein metabolism and interact with several organelles (Welte and Gould, 2017).

## Flavivirus Cellular Cycle Is Intricately Linked to Membrane Lipids

### Lipid Composition of Virions

Flavivirus virions are composed of three structural proteins, all of which interact with lipids. The envelope and membrane proteins form the outer layer of the virion and are anchored in a lipid bilayer membrane. The asymmetric charge distribution of the capsid enables interaction with lipid membranes (Ma et al., 2004) and LDs, the latter being required for virus particle formation (Samsa et al., 2009). Lipid composition of flavivirus virions was only characterized for WNV (Martín-Acebes et al., 2014) and contained a majority of sphingolipids and PLs. Among PLs, PC was the most abundant type, followed by PS and plasmalogen-PC. PE, plasmalogen-PE, lysoPE, and lysoPC were also present but in lower quantities. While there is an abundance of cylindrical lipids such as sphingolipids and PC, integration of the transmembrane envelope and membrane proteins is likely responsible for the curvature that results in a spherical virus particle. Computational models of DENV lipid envelope indicate an important biophysical robustness, characterized by a slow lipid diffusion (Reddy and Sansom, 2016).

### Structural Lipids in Virus Attachment and Entry

**FIGURE 7:**
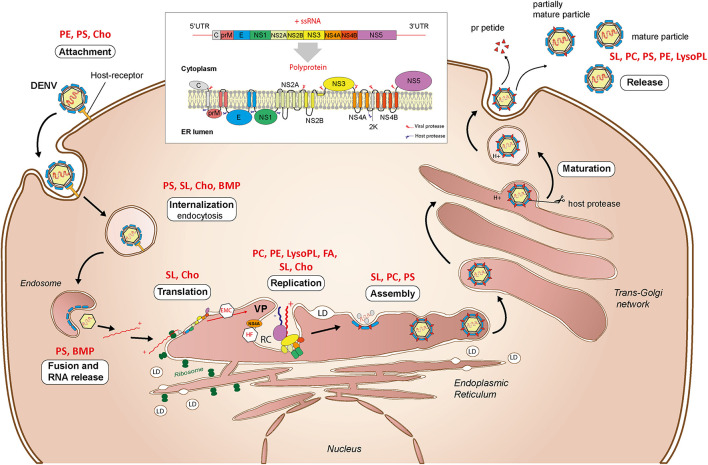
Interactions with lipids during the flavivirus cellular cycle. Attachment, internalization, translation, replication, assembly, maturation, and virus particle release are depicted. Structural lipids involved in the different stages as determined in uninfected cells are indicated in red. UTR, untranslated transcribed region; VP, vesicle packets; RC, replication complex; LD, lipid droplet, ER, endoplasmic reticulum; PE, phosphatidylethanolamine; PS, phosphatidylserine; PC, phosphatidylcholine; SL, sphingolipid; LysoPL, lysophospholipid; Cho, cholesterol; BMP, bis(monoacylglycero)phosphate; FA, fatty acids.

Virus attachment occurs through interactions with cell surface factors, including, but not restricted to, lipids (Figure 7). There exists a variety of receptors in mammalian and mosquito cells, consistent with the ability of flaviviruses to infect a diversity of cells from two different hosts (Hidari and Suzuki, 2011; Cruz-Oliveira et al., 2015). The T-cell immunoglobulin mucin protein domain 1 (TIM) and the tyrosine protein kinase receptor 3-AXL-MER (TAM) families of proteins act as ligands to PS and PE from the viral envelope and promote cell entry (Meertens et al., 2012; Richard et al., 2015). Similarly, human CD300a is a PL receptor and binds directly to PE and PS from DENV particles (Carnec et al., 2016).

After virus adsorption to cell surface, entry occurs mainly by clathrin-dependent endocytosis (Figure 7). As compared to mammals, only clathrin-mediated endocytosis was observed for the four DENV serotypes in mosquito cells (Mosso et al., 2008; Acosta et al., 2011). An invagination in the plasma membrane encloses the virus in a clathrin-coated vesicle, which is transported inside the cell by a mechanism involving actin filaments (Acosta et al., 2008). During internalization, the vesicle acidification changes the envelope protein conformation and enables fusion of viral and endosomal membranes. The fusion process is PL-dependent, especially for PS and bis(monoacylglycero)phosphate (Zaitseva et al., 2010). The viral genome is eventually released as a ribonucleoprotein into the cytoplasm.

Cholesterol is a crucial lipid during flavivirus entry, playing a role both in the target cell and in the viral particle. Depletion of cellular cholesterol inhibits DENV and JEV entry (Lee et al., 2008), while supplementation of viral particles with cholesterol similarly blocks DENV entry (Carro and Damonte, 2013). Cholesterol is enriched in plasma membrane where it associates with PL and sphingolipids to form gel-phase rafts. Alteration of these rafts may influence presentation of attachment receptors or endocytosis.

### Endoplasmic Reticulum Membrane as a Platform for Translation

Once released, the encapsidated genome is uncoated and recruited to ER-bound ribosomes for translation. The single open reading frame of the genome is translated into a multi-pass transmembrane polyprotein, which is processed by viral and host proteases into the three structural proteins (capsid, pr-Membrane, and Envelope) and seven non-structural proteins (NS1, NS2A, NS2B, NS3, NS4A, NS4B, and NS5) (Barrows et al., 2018; Figure 7). Successful folding and post-translational stability require the ER membrane protein complex (Lin et al., 2019; Ngo et al., 2019), sphingolipid and cholesterol-rich lipid rafts (García Cordero et al., 2014). The translation is intertwined with the ER membrane and several viral proteins remain anchored in the ER after translation. While translation and the transmembrane proteins should affect membrane topology and lipid composition, little is known about their impacts on PL reconfiguration.

### Endomembranes at the Heart of Genome Replication

To initiate genome replication, a negative single-strand RNA antigenome is produced from the entering positive-strand genome and then used as a template to duplicate the positive-strand genome (Figure 7). Replication complexes formed by ER invaginations enable genome replication and isolation from host defenses. Structures of DENV replication complex have been characterized in human and mosquito cells (Welsch et al., 2009; Junjhon et al., 2014). In mammalian cells, membrane alterations form several structures: convoluted membranes, double-membrane vesicles, tubular structures, and vesicle packets. Except for convoluted membranes, all the distinct structures were observed in mosquito cells. These different structures are connected by pores, probably to enable transport of building blocks for RNA synthesis and/or release of newly synthetized RNA. Double-membrane vesicles induced by DENV contain NS proteins and dsRNA intermediates, suggesting that they are the site of active RNA synthesis (Miller et al., 2007; Welsch et al., 2009).

Several of the non-structural viral proteins are required to rearrange ER membranes into replication complexes (Schwartz et al., 2002; den Boon and Ahlquist, 2010). Vesicle formation is induced by NS4A through its transmembrane domain (Roosendaal et al., 2006; Miller et al., 2007) and cleavage of its 2K peptide induces membrane arrangement (Miller et al., 2007). Additionally, NS4A triggers rearrangement and phosphorylation of vimentin filaments to support DENV replication complex (Teo and Chu, 2014). NS3 has helicase activity to unfold dsRNA during RNA synthesis, while NS5 has both RNA-dependent-RNA polymerase and methyltransferase activity used to synthetize and cap RNA, respectively. ER-anchored NS4B binds the NS3/NS2B complex which together with NS5 supports replication. NS2A transmembrane protein is also essential for RNA synthesis and is involved in the replication complex organization (Xie et al., 2014).

### Assembly and Maturation Through Endomembrane Network

Flavivirus assembly occurs within the ER membrane (Figure 7). The capsid protein binds the positive-strand genome at multiple sites to fold and tightly package viral RNA (Pong et al., 2011). Encapsidated RNA is probably released from replication complexes through the existing pores, while the mechanism of transport to assembly sites remains unclear. During assembly, ER-anchored membrane and envelope proteins assemble around the capsid, forming a lipid bilayer (Tan et al., 2020). Importantly, the capsid decorates LDs originating from the ER and harnesses them for assembly (Samsa et al., 2009).

Upon assembly, flaviviruses form immature particles characterized by spikes of trimeric pr-membrane, which is the membrane protein precursor, and envelope proteins (Zhang et al., 2003). Maturation occurs through Golgi and *trans-*Golgi networks and requires an acidic environment (Oliveira et al., 2017; Figure 7). Lower pH induces molecular rearrangement of pr-membrane and envelope proteins to expose pr-membrane. Host furin protease then cleaves pr from pr-membrane, maintaining the membrane protein in the mature virion. The presence of pr in viral particles during the maturation might prevent the fusion of the viral envelope with the *trans-*Golgi network (Yu et al., 2009). Immature or partially immature virions with altered infectivity are also produced.

### Exocytosis of Virus Particles

The contents of endomembrane vesicles, i.e., virions and pr peptides, are released into the extracellular space by exocytosis through interaction with the plasma membrane (Figure 7). Little is known about the mechanism of flavivirus egress (Barrows et al., 2018). However, unlike replication and assembly sites in the ER that contain multiple flaviviral particles (Welsch et al., 2009), secretory vesicles usually contain individual flaviviral particles, which are then released individually (Burlaud-Gaillard et al., 2014).

## Flaviviruses Subdue Phospholipids in Mosquitoes

### Specificity of Lipid Metabolism in Mosquitoes

The lipid metabolism pathways are well-conserved between mammals and insects. Fatty acid, PL and glycerolipid biosyntheses are similar except for differences in numbers of enzyme isoforms (Gondim et al., 2018). For instance, while mammals have two acetyl-CoA carboxylases involved in fatty acid metabolism, insects only have one (Alabaster et al., 2011). Similarity in enzyme subcellular localizations, membrane and endomembrane compositions also suggest a certain homology in lipid biosyntheses (Butters and Hughes, 1981). *Aedes* mosquito cells contain an abundance of PC and PE and the same PL categories as in mammals (Townsend et al., 1972; Jenkin et al., 1975). However, while PC is the main PL in mammalian cells, *Aedes* mosquito cells contain a majority of PE (Luukkonen et al., 1973; Jenkin et al., 1975; Dawaliby et al., 2016), and other substantial differences in lipid metabolism between insects and mammals exist (Canavoso et al., 2001).

**FIGURE 8:**
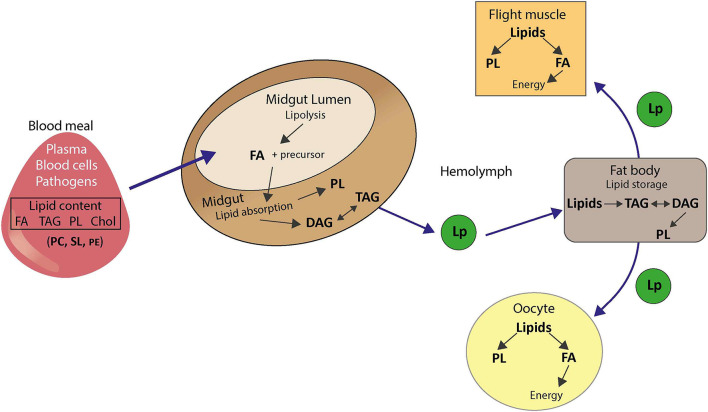
Lipid metabolism in mosquitoes. FA, fatty acid; TAG, triacylglycerol; DAG, diacylglycerol; PL, phospholipid; Cho, cholesterol; PC, phosphatidylcholine; PE, phosphatidylethanolamine; SL, sphingolipid; Lp, lipophorin.

Insects are auxotrophic for cholesterol and must obtain it as well as essential fatty acids from their diet (Figure 8). For mosquitoes, ingested blood is an important source of lipids as it contains free fatty acids, triacylglycerol, cholesterol, and cell-associated PLs. In whole human blood, the most abundant fatty acids are C16:0, C18:1, and C18:2, while PC represents 70–72% of PL in the plasma, 30–36% in erythrocytes, 35–40% in thrombocytes (Hodson et al., 2008). Following blood feeding, midgut lipases and phospholipases catabolize lipids and PLs, respectively, to generate fatty acids. Fatty acids are then absorbed by midgut cells and integrated in the PA pathway to produce PL, triacylglycerol and DAG. Fatty acids can also be synthesized from glucose and amino acids, which is significant given the mosquito mixed diet on nectar sugar and high-protein blood. Lipids are transported in *Aedes aegypti* mosquitoes as TAG in association with the insect lipoprotein called lipophorin, which shuttles lipids to fat body for conversion in triacylglycerol for lipid storage. Stored lipids can be mobilized using the reusable lipophorin shuttle and delivered to targeted tissues for energy or metabolic processes (Gondim et al., 2018). Interestingly, lipophorin is also involved in the immune response and is regulated upon infection (Cheon et al., 2006).

It should be noted that mosquito blood feeding is usually carried out multiple times to reach repletion (Farjana and Tuno, 2013). These multiple blood intakes can successively modify mosquito lipid metabolism. Moreover, blood composition, and more specifically the fatty acid composition of lipids, varies with diet, age, gender, genetic background, and health status (Hodson et al., 2008). As the blood source is an important parameter for the mosquito lipid profile, blood-induced metabolic changes vary among host donors (Kaczmarek et al., 2021). A lipidome study from field-collected mosquitoes is likely to result in high variability, which could explain part of the variation in mosquito vector competence.

### Phospholipid Reconfiguration

Recent studies, including ours, showed that DENV perturb lipid composition in *Aedes* cell lines, midguts, and whole mosquitoes (Perera et al., 2012; Chotiwan et al., 2018; Vial et al., 2019, 2020). Unfortunately, to our knowledge there is no study with other flavivirus species in mosquitoes. While studies at different tissue levels are complementary, variations in metabolic changes between cells and mosquitoes were observed. In *Ae. aegypti* mosquito cell line, DENV infection decreased lipid intermediates such as fatty acid and monoacylglycerol, and increased concentrations in unsaturated PC and PS species at 48 h post infection (Vial et al., 2019). In *Aedes albopictus* cell line, sphingolipids, PC, lysoPC, lysoPE increased at 36 h post-infection in infected cells and in endomembrane fractions containing DENV replication complexes (Perera et al., 2012). A majority of upregulated PC species had unsaturated fatty acyl chains, likely synthetized from PL remodeling (Wang and Tontonoz, 2019), which contributes to PL recycling by incorporating polyunsaturated fatty acids. Interestingly, lysoPLs, resulting from PL hydrolysis, were highly increased early in infected cells (Chotiwan et al., 2018; Vial et al., 2019). Because lysoPLs are the first step in PL remodeling, this is consistent with PL remodeling playing a role in PL reconfiguration during infection. Of note, PE, which represents the majority of PL in insects and is involved in membrane curvature, was not regulated in global cell extracts and only increased in endomembrane fractions (Perera et al., 2012). Such discrepancy between whole cells and subcellular fractions reveals that infection-induced regulation is orchestrated at a fine scale. Overall, these few studies of lipids in cells evidence that flavivirus infection induces a complex reconfiguration of lipid membrane metabolism.

Dengue virus infection in *Ae. aegypti* midgut also modulated membrane lipids by upregulating PC, PE, PS, PG, lysoPL, lysoPI, mono-, di-, triacylglycerols, and sphingolipids (Chotiwan et al., 2018). In another study, our group observed an elevation of lipid intermediates, PE and PC in infected midguts (Vial et al., 2019), confirming that DENV infection modulates PLs. In the same line of thoughts, our study revealed an early increase in PA, the central intermediate in PL biogenesis (Vial et al., 2019). Inversely, anionic PS had a different behavior in our study as the previous one, as we observed a decrease. Differences in virus and mosquito genetics and physiological status (i.e., nutrition, temperature…) may result in metabolic differences. In the sole study with whole mosquitoes, we observed an increase in several species of PLs, especially lysoPL, early in infection, followed by a decrease as infection progressed (Vial et al., 2019). In conclusion, strong modulation of lipids with functions in architecture and expansion of membranes is observed in DENV-infected mosquitoes.

### Regulation of *de novo* Phospholipid Biogenesis

**FIGURE 9:**
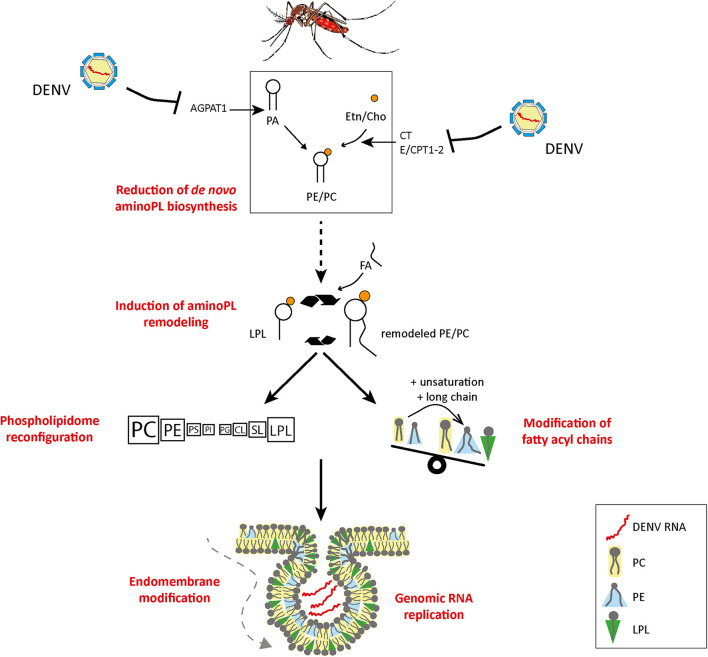
Model of DENV-induced phospholipid reconfiguration for viral replication. We propose that flaviviruses reconfigure PLs by inhibiting *de novo* PL biosynthesis via enzyme regulation and by inducing PL remodeling to enable the formation of replication complexes. AGPAT, 1-acyl-sn-glycerol-3-phosphate O-acyltransferases; CT, triphosphate:phosphocholine cytidylyltransferase; E/CPT1-2, DAG:CDP-ethanolamine/choline ethanolamine/cholinephosphotransferase; PC, phosphatidylcholine; PE, phosphatidylethanolamine; PS, phosphatidylserine; PI, phosphatidylinositol; PG, phosphatidylglycerol; CL, cardiolipin; SL, sphingolipid; LPL, lysophospholipid; FA, fatty acid; Etn, ethanolamine; Cho, choline; AminoPL, aminophospholipid.

Phospholipid reconfiguration can occur either by regulating *de novo* PL biogenesis or PL remodeling, each pathway generating a different set of PLs with different biochemical properties. *De novo* PLs are generally less unsaturated than remodeled PLs (Barelli and Antonny, 2016). Several lines of evidence indicate that *de novo* PLs are not favorable to virus multiplication (Figure 9 and Table 1). We have shown that inhibition of several enzymes involved in *de novo* PL biogenesis promotes DENV infection in mosquito cells (Vial et al., 2020). Likewise, we showed that depletion of AGPAT1 enzyme involved in the synthesis of PA, the precursor for all *de novo* PLs, increased virus load (Vial et al., 2019). Mosquito AGPATs were phylogenetically characterized based on functional motifs defining substrates affinity and acyltransferase activity (Yamashita et al., 2007) as compared to human AGPATs (Vial et al., 2019). Interestingly, DENV reduced mosquito AGPAT1 expression *in vitro* and *in vivo* throughout the infection cycle, whereas another AGPAT isoform with similar function in PA generation was not regulated. Altogether, these results suggest that flaviviruses specifically regulate enzymes of PL biogenesis to promote viral multiplication.

**TABLE 1:** Functions of lipid-related mosquito factors in flavivirus infection.

Pathway	Factor	Effect on flavivirus multiplication	Lipid species involved	References
*De novo* PL	FAS	Proviral	PL, SL, FA and intermediates	Perera et al., 2012
	AGPAT1	Antiviral	PL and LysoPL	Vial et al., 2019
	Kennedy pathway (*CT, ECPT1-2*)	Antiviral	AminoPL and lysoPL	Vial et al., 2020
	Ethanolamine	Antiviral	AminoPL and LysoPL	Vial et al., 2020
	CL synthase	Proviral	CL	Koh et al., 2020
PL remodeling	PLA2	Proviral	LysoPC	Liebscher et al., 2018
Other lipid pathways	SL desaturase	Proviral	SL	Chotiwan et al., 2018
	Lipid droplets	Proviral	Likely TAG, FA, PL, sterol esters	Barletta et al., 2016
	Lipophorin receptor (LpR)	Antiviral	TAG	Koh et al., 2020
	LRP-1	Antiviral	Cholesterol	Tree et al., 2019
	SREBP	Proviral	Unknown	Raquin et al., 2017

*FAS, fatty acid synthase; AGPAT1, 1-acyl-sn-glycerol-3-phosphate O-acyltransferase 1; CT, triphosphate:phosphocholine cytidylyltransferase; ECPT, DAG:CDP-ethanolamine/choline ethanolamine/cholinephosphotransferase; Etn, Ethanolamine; PLA2, Phospholipase A2; CL, cardiolipin synthase; DEGS, sphingolipid Δ-4 desaturase; LD, lipid droplet; LpR, lipophorin receptor; LRP-1, low-density lipoprotein receptor-related protein 1; SREBP, sterol regulatory element-binding protein.*

Enzyme expression can be regulated either directly by transcription factor or indirectly by alteration of PL content, ER stress response or endoplasmic reticulum topology. Transcriptionally, multiple enzymes involved in lipid metabolism are regulated by the sterol regulatory element binding proteins (SREBP; Coleman and Lee, 2004; Karasawa et al., 2019). SREBP chemical inhibition was shown to reduce viral replication (Merino-Ramos et al., 2017), suggesting a proviral function for SREBP lipid regulation. However, DENV-induced ER membrane rearrangement was not linked to SREBP signaling (Peña and Harris, 2012). Instead, ER modification was dependent on viral protein expression and lipid reabsorption into the ER. Those results suggest that DENV alteration of PL metabolism is not mediated by SREBP signaling but instead is directly related to recycling of PL species and viral proteins.

Introgression of the viral proteins into the ER membrane should alter membrane topology, which in turn regulates transmembrane protein function (Bogdanov et al., 2014). For instance, stresses in the lipid bilayer disturb ER-resident proteins (Shyu et al., 2019) and results in accumulation of unfolded or misfolded proteins, a phenomenon known as the unfolded protein response (Gentile et al., 2010). Subsequently, ER-induced stress disrupts lipid metabolism, such as glycerolipid and cholesterol biosynthesis (Werstuck et al., 2001). All these suggest that ER membrane stress imposed by infectious processes might affect enzyme expression and activity by altering protein anchorage, acyl substrate specificity or transferase activity. These changes to the lipid metabolism may then regulate PL production.

### Induction of Phospholipid Remodeling

The fatty acyl chain of *de novo* PL can be modified by PL remodeling through the Lands cycle (Figure 5). Remodeling enables cells to produce new PL species with different biochemical properties to support membrane maintenance and diversity (Wang and Tontonoz, 2019). There is a negative cross-talk between *de novo* PL biogenesis and PL remodeling, whereby activation of one inhibits the other. Previous observation that *de novo* PL production is inactivated by DENV suggests that PL remodeling is induced by infection (Figure 9; Vial et al., 2020). To demonstrate activation of PL remodeling, we developed isotope tracing of PL in mosquito cells. Cells were supplemented with a labeled precursor of *de novo* PLs. We provided the label enough time before infection so that PLs but not lysoPLs were labeled. In these conditions, production of lysoPLs indicated PL deacylation and increase in non-labeled PLs was a consequence of PL remodeling. Inversely, increase in labeled PLs showed activation of *de novo* PL biosynthesis. Upon infection, we observed an early production of lysoPLs and an increase in non-labeled PLs, demonstrating induction of the PL remodeling cycle upon infection. However, we subsequently observed an increase in isotope labeled PLs, showing activation of *de novo* PL biosynthesis. Together, this indicates that DENV infection induces PL remodeling and, later on, *de novo* PL biogenesis, possibly to replenish PL depleted by remodeling.

Interestingly, in mosquito cells, WNV was shown to increase PLA2 activity (Table 1; Liebscher et al., 2018). PLA2 initiates PL remodeling by cleaving PL sn2 acyl to produce lysoPLs. Furthermore, the same study showed that lysoPCs were recruited to replication complexes and required for proper membrane curvature in mammalian cells. In a different study, fatty acid synthase enzyme involved in lipogenesis was required during DENV infection in mammalian cells (Perera et al., 2012). DENV NS3 recruited the fatty acid synthase to replication sites and modified the enzyme activity to stimulate malonyl-CoA incorporation into fatty acids (Heaton et al., 2010). This altered activity produces more palmitate, which is then used for complex lipid biogenesis, including polyunsaturated fatty acids. The newly synthesized fatty acids could serve to reacylate the lysoPLs and complete PL remodeling cycle, thereby diversifying PL composition (The British Nutrition Foundation, 1992). Taken together, we propose a model whereby PL remodeling is induced by flavivirus infection to generate a membrane architecture conducive to the formation of replication complexes (Figure 9).

### Functions of Other Lipid Pathways in Flavivirus Infection

Dengue virus infection in mosquitoes also relies on other lipids that PLs (Table 1). Sphingolipids are lipid membranes and contribute to DENV multiplication. Chemical inhibition of sphingolipid Δ-4 desaturase, which synthesizes ceramide, reduced DENV multiplication (Chotiwan et al., 2018). Storage of triacylglycerol in LDs is increased upon DENV infection in cells and mosquito midguts (Barletta et al., 2016; Koh et al., 2020). DENV NS4A interacts with Ancient Ubiquitous Protein 1 (AUP1), a LD-associated acyltransferase, to activate LD lipophagy (Zhang et al., 2018). Cholesterol pathway is involved in DENV multiplication (Raquin et al., 2017; Tree et al., 2019). Low-density lipoprotein receptor-related protein 1 (LRP-1) involved in cholesterol regulation was downregulated by infection (Tree et al., 2019), while LRP-1 inhibition increased intracellular cholesterol quantity and promoted DENV infection. A lipophorin receptor (LpR), named as a very low density lipophorin receptor was also down-regulated by DENV infection, although LpR knockdown did not affect DENV replication (Koh et al., 2020). CL synthase involved in CL biogenesis, a mitochondrial PL, was downregulated in DENV-infected *Ae. aegypti*, although CL synthase promoted infection (Koh et al., 2020). CL alteration may disrupt mitochondrial membrane integrity, modifying intrinsic properties of mitochondria, such as energy metabolism and apoptosis regulation (Gonzalvez and Gottlieb, 2007). Altogether, the whole lipid environment is engaged by flaviviruses to enable viral multiplication.

### Phospholipids in Innate Immunity and Signaling

Mosquitoes mount a potent antiviral response to infection through multiple immune pathways. The Janus kinase/signal transducers and transcription activator pathway (JAK-STAT), immune deficiency pathway (IMD), the Toll pathway, the Jun-N-terminal Kinase (JNK) pathway, and the RNA interference pathway (RNAi) have all been shown to reduce flavivirus infection (Sim et al., 2014; Chowdhury et al., 2020). Interestingly, there exist bridges in signaling pathways between immune responses and lipid metabolism in mosquitoes. Toll pathway activation by bacteria, fungi or parasites induces expression of fat body genes related to lipid metabolism (Cheon et al., 2006). DENV infection in *Ae. aegypti* upregulates genes associated with LD biosynthesis, while activation of either Toll or IMD pathways increase LD number (Barletta et al., 2016). The interaction between immune response and lipid metabolism certainly deserves further studies.

Reconfiguration of the lipidome upon infection in mosquitoes may also modify lipid-mediated signaling. Prostaglandins that regulate immunity and inflammation (Harris et al., 2002) are regulated by bacterial and parasite infection in mosquitoes (Muñoz et al., 2008). Prostaglandins are produced from C20 polyunsaturated fatty acids, which can be derived from PL hydrolysis via PLA2 cleavage during PL remodeling.

### Identification of Antiviral Targets

Because of the lack of treatment and of effective vaccines against all flaviviruses, there is a great need for new strategies to block flavivirus transmission. Targeting branches of lipid metabolism that are required for virus infection may hold the key to novel antiviral tools. While lipid-related anti-flaviviral strategies have already been reviewed (Martín-Acebes et al., 2016), targeting of PL remains limited when applied to mosquitoes. In mammalian cells, the most preponderant PI (20:4/18:0) is composed of one acyl chain of arachidonic acid and one of stearic acid and has anti-DENV activity (Sanaki et al., 2019). The PI acts by suppressing DENV-induced cytokine inflammation.

As PL remodeling promotes DENV infection in mosquitoes (Vial et al., 2020) whereas *de novo* PL biogenesis reduces virus load, disrupting PL biosynthesis may hinder viral multiplication. Induction of *de novo* PL biogenesis by supplementing the blood on which mosquitoes feed with a *de novo* PL precursor reduced mosquito midgut infection (Vial et al., 2020). Harnessing this knowledge, the PL precursor could be delivered through sugar feeding to reduce infection. Similarly, as mosquitoes can derive ethanolamine from PE, *de novo* PL biogenesis could be activated by exogenous supply of PE. Moreover, flavivirus infection significantly modifies blood lipid composition (Cui et al., 2013; Lima et al., 2019), which then influences infection onset in mosquito midguts (Vial et al., 2020). Chemical alteration of blood lipids from patients may help disrupt the flavivirus transmission cycle.

Lipid-targeted therapeutics have been identified for other viruses. Influenza virus infection is blocked by the fatty acid docosahexaenoic acid, which limits transport of viral transcripts (Morita et al., 2013). Supplementation with PL such as PG and PI also suppresses influenza virus and syncytial virus infection. Antibody targeting of PS was applied to treat arenavirus and cytomegalovirus infection in animal models (Soares et al., 2008). Antibodies aimed at PL to induce an immune response were similarly applied to neutralize HIV-1 in peripheral blood mononuclear cells (Moody et al., 2010).

## Conclusion

Our understanding of the lipid interactions between mosquitoes and flaviviruses has expanded in recent years, thanks to innovative *in vitro* and *in vivo* metabolomic approaches. However, there remain much to understand. The importance of PL and its reconfiguration for DENV infection in mosquitoes is well established now but studies with multiple flavivirus species are required to determine the conservation of these lipid needs. Further mechanistic analyses will also help identify the PL species involved and understand how flaviviruses alter their concentrations. In the end, characterized PLs will provide ideal targets for novel transmission blocking strategies.

## Author Contributions

TV and JP wrote the original draft. TV, DM, GM, and JP edited the manuscript. All authors contributed to the article and approved the submitted version.

## Conflict of Interest

The authors declare that the research was conducted in the absence of any commercial or financial relationships that could be construed as a potential conflict of interest.

## Publisher’s Note

All claims expressed in this article are solely those of the authors and do not necessarily represent those of their affiliated organizations, or those of the publisher, the editors and the reviewers. Any product that may be evaluated in this article, or claim that may be made by its manufacturer, is not guaranteed or endorsed by the publisher.
